# Impaired Cellular Energy Metabolism Contributes to Duck-Enteritis-Virus-Induced Autophagy via the AMPK–TSC2–MTOR Signaling Pathway

**DOI:** 10.3389/fcimb.2017.00423

**Published:** 2017-09-26

**Authors:** Haichang Yin, Lili Zhao, Siqi Li, Lijing Xu, Yiping Wang, Hongyan Chen

**Affiliations:** State Key Laboratory of Veterinary Biotechnology, Heilongjiang Provincial Key Laboratory of Laboratory Animal and Comparative Medicine, Harbin Veterinary Research Institute, the Chinese Academy of Agriculture Sciences, Harbin, China

**Keywords:** duck enteritis virus, duck embryo fibroblasts, autophagy, cellular energy metabolism, AMPK–TSC2–MTOR signaling pathway

## Abstract

Duck enteritis virus (DEV) is a large, complex double-stranded DNA virus that induces duck embryo fibroblast (DEF) cells autophagy, which is beneficial to its own replication, but the mechanism has not been described. In this study, we showed that impaired cell energy metabolism is involved in DEV-induced autophagy, whereby ATP synthesis is disrupted in cells after DEV infection, which causes metabolic stress and activation of autophagy. Methyl pyruvate (MP) inhibited conversion of LC3I to LC3II and accumulation of GFP-LC3, which could reverse the energy loss caused by DEV infection. Inhibition of DEV replication by MP confirmed the above view. We found that infection with DEV activated the metabolic regulator 5′ AMP-activated kinase (AMPK) and inhibited activity of mechanistic target of rapamycin (mTOR). In the cases where AMPK expression was inhibited, the LC3-I conversion to LC3-II ratio was decreased, as was GFP-LC3 point and DEV replication; in addition, inhibition of p-mTOR showed a reverse trend. We also found that tuberous sclerosis (TSC) 2 was a key mediator between AMPK and mTOR through activation by phosphorylation. siRNA targeting TSC2 was transfected to reverse the inhibition of mTOR, and down-regulate autophagy level and DEV replication, but AMPK expression was not changed, while siRNA targeting AMPK inhibited activation of TSC2. In conclusion, our findings indicate that energy metabolism in cell damage induced by DEV contributes to autophagy via the AMPK–TSC2–MTOR signaling pathway, which provides a new perspective for DEV and host interactions.

## Introduction

Duck viral enteritis (DVE) is characterized by vascular injury, bleeding, gastrointestinal mucosal erosion, lymphoid organ damage, and substantive organ-degenerative lesions (Wang et al., 2013). Its rapid spread, high morbidity and mortality bring huge economic losses to the commercial duck industry. The pathogen of DVE is duck enteritis virus (DEV). DEV has a double-stranded DNA genome of ~160 kb, which includes a long unique area (UL), short unique area (US), repetitive sequence at both ends of the US (TRS), and a repetitive sequence in the middle (IRS).The arrangement of the genome is UL–IRS–US–TRS (Gardner et al., 1993; Li et al., 2009). Research on the molecular biology of DEV has lagged behind that of other viruses. Therefore, little is known about its genome, proteome and pathogenic mechanism. This leads to difficulties in the prevention and control of DVE.

Autophagy is a widespread self-eating phenomenon in eukaryotic cells that takes place through the degradation of long-lived proteins and damaged organelles. It is an important repair pathway that recycles nutrients to enable survival under stress conditions (Codogno and Meijer, 2005). According to type of substrate, transport processes and regulatory mechanism, autophagy can be divided into macroautophagy, small autophagy and molecular-chaperone-mediated autophagy (Wang and Klionsky, 2003). Macroautophagy refers to the process by which double-layer membranes from the endoplasmic reticulum fuse with lysosomes to form autophagosomes, followed by degradation of their contents. There is strong evidence that autophagy is an innate immune defense mechanism against external pathogenic microorganisms; however, many pathogens use the autophagy response to survive and replicate (Deretic, 2010). Our preliminary research demonstrated that DEV induced autophagy, which facilitates its own replication (Yin et al., 2017), but the mechanism is still unknown.

Extracellular stress signals, such as hunger, growth factor defects, endoplasmic reticulum stress and pathogen invasion, can induce cell autophagy. Metabolic stress can also be an activator of autophagy. Autophagy is required for cells which do not have sufficient energy to survive, and the mechanism involved in the signaling pathway for metabolically activated autophagy has been well studied. In mammalian cells, ATP levels are detected by AMP-activated protein kinase(AMPK). When the balance of ATP/AMP is destroyed, upstream liver kinase (LK)B1 activates AMPK. Activation of AMPK can lead to tuberous sclerosis (TSC)1/2 complex activation, and activity of mammalian target of rapamycin (mTOR) can be inhibited by the complex activated through Rheb (Ras homolog enriched in brain) (Inoki et al., 2003). mTOR-dependent activation of autophagy leads to a higher level of ATP through the nutritional energy cycle. In addition, p27^kip1^, a cell-cycle-dependent protein kinase inhibitor, is activated and phosphorylated by the LKB1–AMPK pathway. This can lead to stagnation of the cell cycle, which can cause living pressure, due to growth factor and nutritional deprivation, which induces autophagy to maintain cell survival (Liang et al., 2007). Similarly, Snf1 (sucrose non-fermenting1), an AMPK homolog in yeast, positively regulates autophagy, which may involve autophagy related 1 (ATG1)-dependent regulation (Wang et al., 2001).

Energy metabolism is not only necessary for cell survival, but is also related to replication of many viruses (Levine, 2005). This means that energy metabolic regulation is at the center of host virus interactions. This interaction can often result in severe metabolic stress. However, it is still unknown whether DEV infection causes energy metabolic in the cell, which is responsible for autophagy activation.

Our findings indicate that energy stress in cells infected with DEV contributes to autophagy via the AMPK–TSC2–MTOR signaling pathway. This research lays a foundation for research on the DEV pathogenic mechanism, and provides drug targets for prevention and control of DVE in the water industry.

## Materials and methods

### Cell, virus, and plasmids

DEF cells were obtained from 9 to 11-day-old specific pathogen-free duck embryos as described previously (Jacolot et al., 2008) and cultured in Dulbecco's modified Eagle's medium (Gibco) supplemented with 5% FBS (Gibco) and antibiotics (0.1 mg/ml streptomycin and 0.1 mg/ml penicillin) at 37°C in 5% CO_2_. DEV CSC strain was purchased from China Institute of Veterinary Drug Control. Mouse monoclonal antibody against glycoprotein B (gB) was kept in our laboratory.

To construct a GFP-LC3 recombination plasmid, the duck *LC3B* gene was amplified from DEF cells with primers LC3F 5′-ATGCAACCGCCTCTG-3′ and LC3R 5′-TCGCGTTGGAAGGCAAATC-3′, according to the GenBank sequence for duck LC3B (NW_004676873.1), and cloned into pEGFP-C1 plasmid prepared in our laboratory, to express LC3 with the EGFP protein at its N terminus.

### Virus infection and drug treatment

DEV of m.o.i 1 was removed after 2 h infection at 37°C. DEF cells were washed three times with sterile PBS (pH7.4) and maintained in 2% FBS in culture medium until samples were harvested. DEF cells were then cultured in fresh media in the absence or presence of the same drug as for pretreatment for the indicated times. Chemicals and their optimal concentrations used in this experiment included 10 mM MP (Sigma), and 5 μM Compound C (Merck–Millipore). At 48, 60, and 72 hpi, DEF cells were collected for subsequent experiments.

### SDS-PAGE and western blotting

Drug-treated, siRNA-transfected and virus-infected cellular proteins were extracted as follows: cell protein was extracted using IP lysis buffer (Beyotime, Jiangsu, China) and protease inhibitor PMSF (Beyotime). Protein samples with 5 × loading buffer were boiled for 10 min, analyzed by 12% SDS-PAGE, and transferred onto nitrocellulose membranes (GE Healthcare Life Sciences, Little Chalfont, UK). The membranes were blocked with 3% BSA (Sigma) for 2 h at room temperature, and then incubated with primary antibody for 2 h at room temperature: rabbit anti-LC3B (L7543), rabbit anti-p-mTOR (SAB4504476), mouse anti-β -actin (A1978), rabbit anti-mTOR (SAB2701842), rabbit anti-pTSC2 (SAB4504003), rabbit anti-TSC2 (SAB4503037) (Sigma–Aldrich), rabbit anti-p-AMPK (44-1150G) or rabbit anti-AMPK (AHO1332) antibody (Thermo Scientific). The membranes were incubated with IRDye 800 CW goat anti-mouse IgG or goat anti-rabbit IgG as secondary antibodies (LI-COR Biosciences) for 1 h at room temperature. Detection was carried out using an Odyssey Infrared Fluorescence Scanning Imaging System (LI-COR Biosciences).

### TEM

TEM observation of autophagy was carried out as described previously (Alexander et al., 2007). DEF cells in 25-cm^2^ flasks were collected at 48 h after DEV infection, with mock-infected cells as controls. Ultrathin sections were viewed under an H-7650 transmission electron microscope (Hitachi).

### Confocal fluorescence microscopy

For the detection of autophagosomes, DEF cells were transfected with 2.5 μg GFP-LC3 plasmid using the Calcium Phosphate Transfection Kit (Invitrogen) when cells were grown to 70–80% confluence in culture dishes, at 24 hpi. Drug-treated, siRNA-transfected or virus-infected DEF cells were fixed in absolute ethanol for 30 min, and nuclei were stained by DAPI (Sigma). The green fluorescence dots of GFP-LC3 were observed using a Leica SP2 confocal microscopy system (Leica Microsystems).

### RNA interference of genes *AMPK* and *TSC2*

To study the effects of cell autophagy on viral replication, siRNAs targeting *AMPK* and *TSC2* were synthesized (Shanghai GenePharma). The sequence of siRNA targeting *AMPK* was: AMPK-1#, GCAGGUCCAGAAGUAGAUATT(sense) and UAUCUACUUCUGGACCUGCTT(antisense); AMPK-2#, GCCAUUCUUGGUAGCCGAATT (sense), UUCGGCUACCAAGAAUGGCTT(antisense). AMPK-3#, GCACAUUAGGCUUCAUAUATT(sense), UAUAUGAAGCCUAAUGUGCTT(antisense). The sequence of siRNA targeting *TSC2* was: TSC2-1#, GCUGCUAUCUGGAAGACUATT(sense), and UAGUCUUCCAGAUAGCAGCTT(antisense); TSC2-2#, GCCACUAUAUGUACUCGUATT(sense), and UACGAGUACAUAUAGUGGCTT(antisense); TSC2-3#, GCAAAUGGCAGAGAAGUAATT(sense), UUACUUCUCUGCCAUUUGCTT(antisense). Six-well plates were transfected with siRNAs and negative control RNA (siNC) using transfection reagents for 24 h and then infected with DEV. Cell samples were collected at 48 hpi to detect silencing effects and used in subsequent experiments.

### TCID_50_

The cell monolayers were infected with DEV serially diluted from 10^−1^ to 10^−8^ in 96-well plates. Virus was removed after infection for 2 h at 37°C and cells were washed three times with sterile phosphate-buffered saline. DEF cells were maintained in 2% FBS in culture medium at 37°C for a further 72 h. At the final time point, we observed and recorded cytopathological changes, and virus titres were determined according to the Reed–Muench method.

### ATP determination assay

Once the cell culture supernatant was discarded, we added 200 μl lysis buffer to the wells in a six-well plate. Cells were centrifuged 12,000 *g* for 5 min at 4°C and the supernatant was used for subsequent determination of ATP. We then added 100 μl ATP test liquid into inspection wells, which were then incubated for 3–5 min at room temperature, to ensure that all of the background ATP was removed before analysis; 20 μl of the sample was then added to the inspection well, rapid blending, then after at least 2 s, RLU readings were measured using an EnVision Multilabel Reader (Perkin Elmer).

### Statistical analysis

All experimental results are expressed as the mean ± SD. All data were analyzed in three independent experiments. Tukey's test was used for statistical analysis. The difference between two group means is presented as *P* < 0.05 (^*^) and *P* < 0.01 (^**^).

## Results

### DEV infection induces energy metabolism in DEF cells

**Figure 1:**
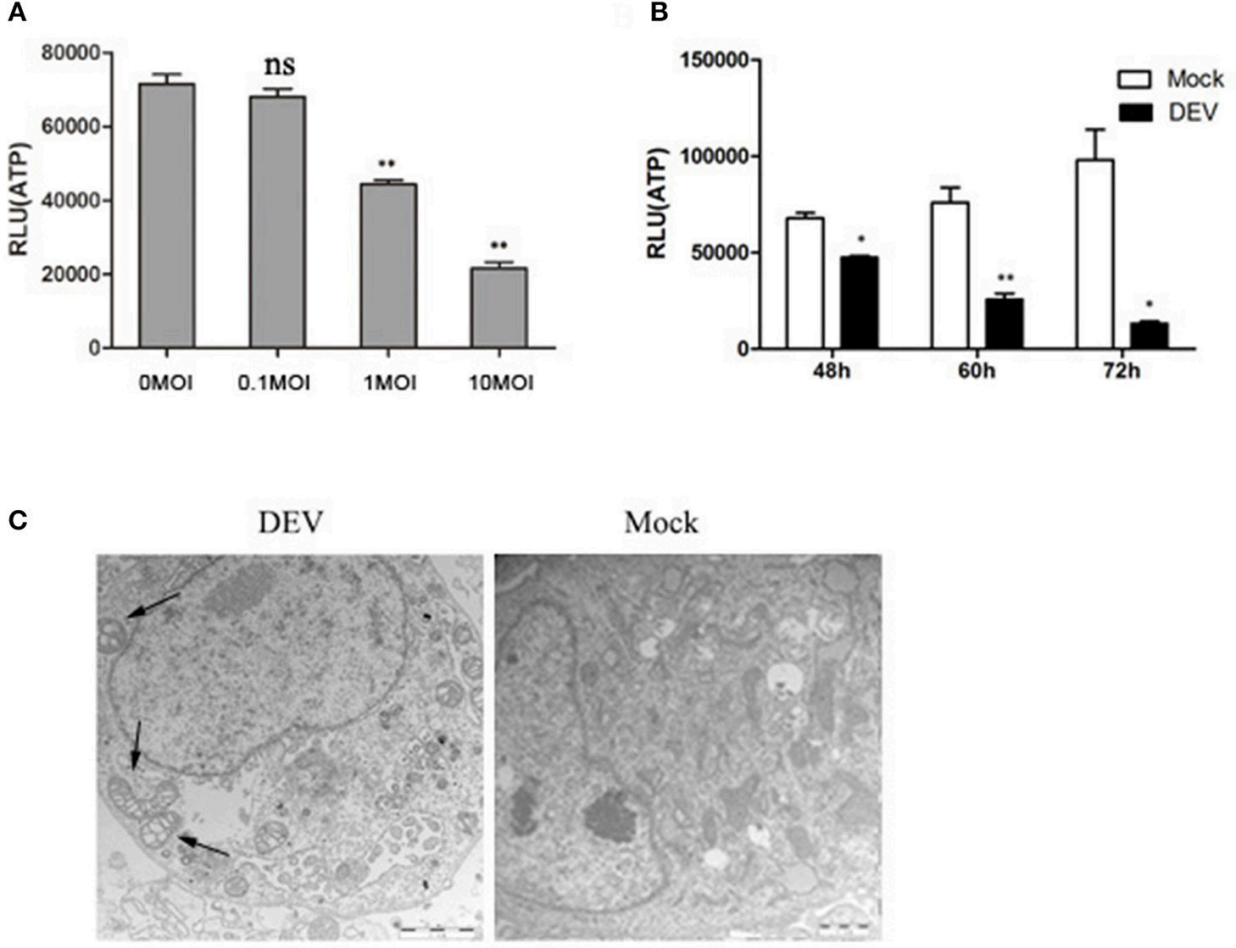
DEV infection induces cell energy deficiency in a dose- and time-dependent manner. DEF cells were infected with DEV at different doses (m.o.i.) **(A)** for 48 hpi or at m.o.i. 1 for 48, 60, and 72 hpi **(B)**, followed by measurement of ATP levels using a Bioluminescence Assay Kit. **(C)** DEV infection promotes morphological alterations in mitochondria in DEFs. TEM of a mock-infected cell showing mitochondria with characteristic electron-dense matrices (arrows); DEV-infected cell presenting swollen mitochondria with loss of electron density in the matrix (arrows). Bar = 1 μm. The difference between two group means is presented as ^*^P < 0.05 and ^**^P < 0.01.

We investigated whether energy metabolism was related to autophagy response during DEV infection. The supply of ATP is an important indicator of cell energy, so ATP levels were measured using the firefly-luciferase-based ATP assay. Changes in ATP levels were indicated by relative light unit (RLU) values, which were measured in three independent repeat tests. ATP levels were reduced in a dose- and time-dependent manner in DEV-infected DEF cells (Figures 1A,B). We also showed that DEV infection had no affect on cell viability (Figure S7). In addition, we counted cell numbers to confirm whether the samples had equal cell viability, to confirm that cellular ATP was indeed decreased in DEV-infected cells. In other words, DEV infection caused energy stress in DEF cells.

We used transmission electron microscopy (TEM) to establish whether the changes in mitochondrial energy metabolism were associated with changes in morphology. DEV-infected DEF cells had reduced electron density in the mitochondrial matrix, but in contrast, mock-infected cells showed mitochondria with characteristic electron-dense matrices (Figure 1C). The original pictures of Figure 1 were shown in Supplementary Materials (Figure S1).

### Energy stress can contribute to DEV-induced autophagy in DEF cells

**Figure 2:**
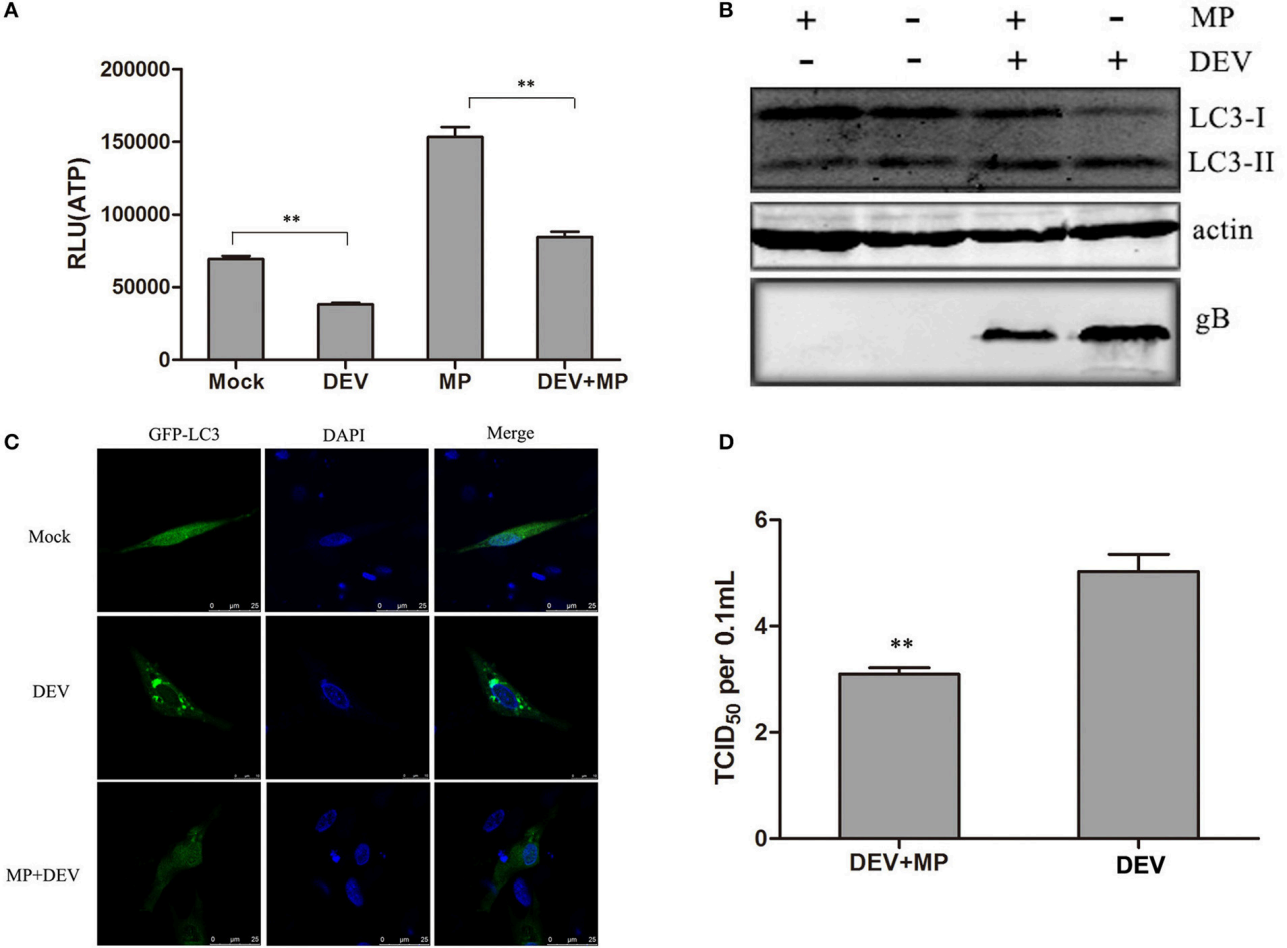
Cellular energy depletion is involved in DEV-induced autophagy. **(A)** Energy was restored by MP treatment. Mock- or DEV-infected DEF cells were treated with 10 mM MP for 36 h or untreated, and the ATP levels were measured. **(B)** Cells were infected and treated, and at 48 hpi, cell samples were analyzed by immunoblotting with antibodies against LC3B, gB, and β -actin. **(C)** Confocal microscopy. DEF cells were transfected with a plasmid expressing GFP-LC3 for 24 h. Autophagosomes were seen as green puncta. The cell nuclei were stained with DAPI. **(D)** Reduction of DEV replication by MP treatment. Cells were pretreated and infected. At 48 hpi, virus titres were measured using the TCID_50_ assay. The difference between two group means is presented as ^**^P < 0.01.

To investigate whether this energy stress can contribute to DEV-induced autophagy, we explored whether consumption of ATP in DEV-infected cells was reversed by methyl pyruvate (MP). MP is a cell-permeable form of pyruvate and can be oxidized in the tricarboxylic acid cycle to produce NADH to fuel electron transport and ATP production (Bhutia et al., 2010). Mock- or DEV-infected DEF cells were treated with or without 10 mM MP for 48 h, and ATP levels were measured using a firefly-luciferase-based ATP assay kit. MP restored ATP production in DEV-infected cells (Figure 2A). The conversion of LC3I to LC3II was examined by western blotting after MP treatment. The changes in autophagy in MP-treated or DEV-infected cells were evaluated by the ratio of LC3II to LC3I. Conversion of LC3I to LC3II clearly increased upon DEV infection when compared with mock-infected cells, suggesting that autophagy was triggered upon DEV infection. LC3II expression was reduced after addition of MP to DEV-infected cells (Figure 2B), suggesting that relief of energy stress by ATP restoration reduced DEV-induced autophagy.

LC3II was also observed by confocal fluorescence microscopy as discrete puncta associated with autophagic vacuoles, and the increase in GFP–LC3 puncta after treatment may reflect increased autophagic activity. As expected, the number of GFP–LC3 puncta decreased dramatically when DEV-infected cells were treated with MP, suggesting that autophagy was inhibited (Figure 2C). Our data demonstrate that DEV-induced disruption of cellular energy contributes to autophagy.

Less DEV glycoprotein B (gB) was observed in MP-treated cells compared with mock-treated cells (Figure 2B). Consistent with this result, DEV titres were significantly reduced in MP-treated cells compared with control cells. TCID_50_ was used to measure the viral titres (Figure 2D). These results also indicate that the MP-restored energy state inhibited DEV-induced autophagy and DEV replication. In addition, we also showed ATP production inhibitor could enhance DEV replication and autophagy (Figure S6). Our study confirms that DEV infection reduces ATP level, resulting in a type of energy stress, followed by induction of autophagy. The original pictures of Figure 2 were shown in Supplementary Materials (Figure S2).

### AMPK–mTOR may be involved in DEV-induced autophagy

**Figure 3:**
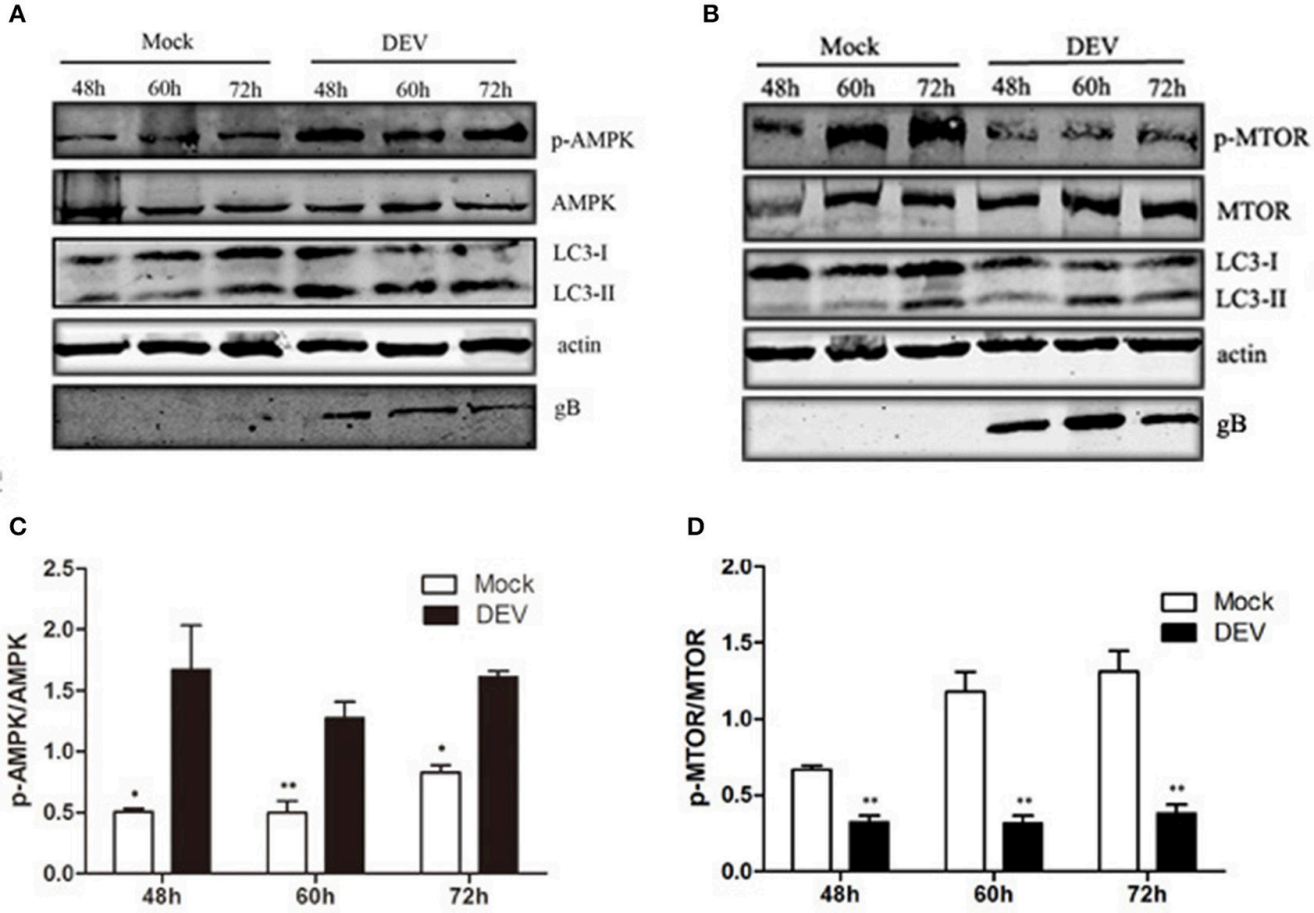
AMPK–mTOR may be involved in DEV-induced autophagy. **(A,B)** DEF cells were infected with DEV. At 48, 60, and 72 hpi, cells were harvested and the activity of AMPK and mTOR was analyzed by western blotting using the indicated antibodies. **(C,D)** Intensity band ratio of p-mTOR to mTOR and p-AMPK to AMPK. The difference between two group means is presented as ^*^P < 0.05 and ^**^P < 0.01.

AMPK is a protein kinase, which is regulated by the energy metabolism in cells. This protein is a key regulator of autophagy (Meley et al., 2006). Lack of energy in cells and changes in AMP level are detected by AMPK, when the AMP/ATP ratio increases, AMPK is activated (Inoki et al., 2003). AMPK also is a key negative regulator of the MTOR pathway (Shang and Wang, 2011). We aimed to determine whether AMPK was involved in DEV-induced autophagy and inhibition of MTOR activity in DEF cells. DEV induced DEF cells autophagy at 48, 60, and 72 h post-infection (hpi), as demonstrated by significantly increased conversion of LC3I to LC3II. DEV increased expression of phosphorylated (p)-AMPK in DEFs cells and increased AMPK activity (Figures 3A,C). DEV also decreased the level of p-mTOR; however, the corresponding expression of LC3II increased (Figures 3B,D). Therefore, it can be speculated that AMPK was involved in mTOR signaling pathways. The original pictures of Figure 3 were shown in Supplementary Materials (Figure S3).

### AMPK regulates DEV-induced autophagy through mTOR

**Figure 4:**
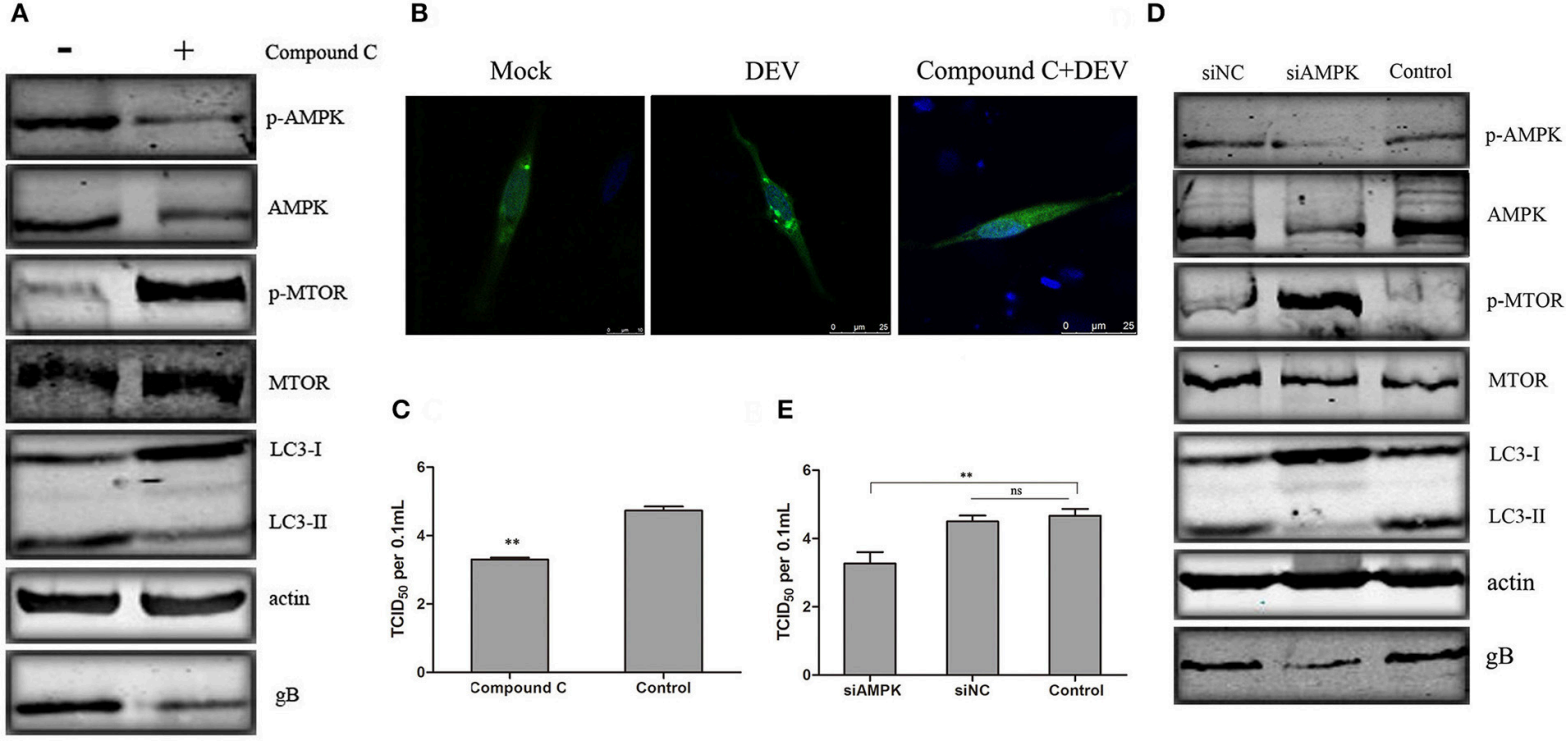
AMPK regulates DEV induced autophagy through mTOR. **(A)** Effects of Compound C treatment on LC3II, phosphorylation and total levels of AMPK and mTOR. Cells were pretreated with Compound C (5 μM) or DMSO (control) for 1 h, followed by DEV adsorption for 2 h. At 48 hpi, the protein levels were measured by western blotting. **(B)** DEF cells transfected with GFP-LC3 for 24 h were treated with 5 μM Compound C or DEV. Formation of GFP-LC3 puncta was analyzed. **(C)** Titers of DEV produced by Compound-C-treated DEF cells. Cells were pretreated and infected. Virus yields are shown as TCID_50_/ml at 48 hpi. **(D)** Knockdown of AMPK affected the activity of mTOR and autophagy in DEV-infected cells. DEF cells were transfected with AMPK-specific or control siRNA, then infected with DEV. At 48 hpi, cells were harvested and western blotting was performed. **(E)** Virus yields in DEF cells transfected with siRNA against AMPK. Virus titres were measured using the TCID_50_ assay. The difference between two group means is presented as ^**^P < 0.01.

We verified the role of AMPK in DEV-induced autophagy. AMPK is activated in DEFcells infected with DEV. We used Compound C, a known AMPK inhibitor, to assess changes in p-mTOR corresponding to inhibition of AMPK. In DEV-infected cells, inhibition of AMPK partly reversed p-mTOR inhibition, and mTOR activity then inhibited autophagy induced by DEV, suggesting that AMPK is a negative regulator of mTOR (Figure 4A). Formation of GFP-LC3 puncta verified above speculation. Compared with uninfected cells, DEV-infected cells had a significant increase in GFP-LC3 puncta, while which was markedly decreased by Compound C (Figure 4B). We previously found that autophagy influenced DEV replication. Here, we found that Compound-C-treated cells decreased expression of DEV gB (Figure 4A), and reduced production of virus particles at 48 hpi (Figure 4C).

To eliminate nonspecific effects of chemical Compound C, three pairs of siRNAs targeting AMPK were designed, and the silencing effect was detected. The effective maximum of three siRNAs targeting AMPK was AMPK-3# (Figure S5). Then compared activity of cells treated with a negative control RNA (siNC) to the cells treated with the siRNA, and we observed a corroborating decrease in mTOR signaling when AMPK were targeted. Expression of LC3II was significantly reduced by AMPK (Figure S8) inhibition, which significantly reduced synthesis of DEV gB (Figure 4D). At the same time, the virus yield was reduced at 36 hpi (Figure 4E). Similar results were obtained in cells treated with another two siRNAs (siAMPK-1# and siAMPK-2#) (Figure S9). Our data suggests that AMPK regulates DEV-induced autophagy through mTOR, and is necessary for DEV replication. The original pictures of Figure 4 were shown in Supplementary Materials (Figure S4).

### TSC2 is involved in AMPK-MTOR signaling pathway mediated DEV-induced autophagy

**Figure 5:**
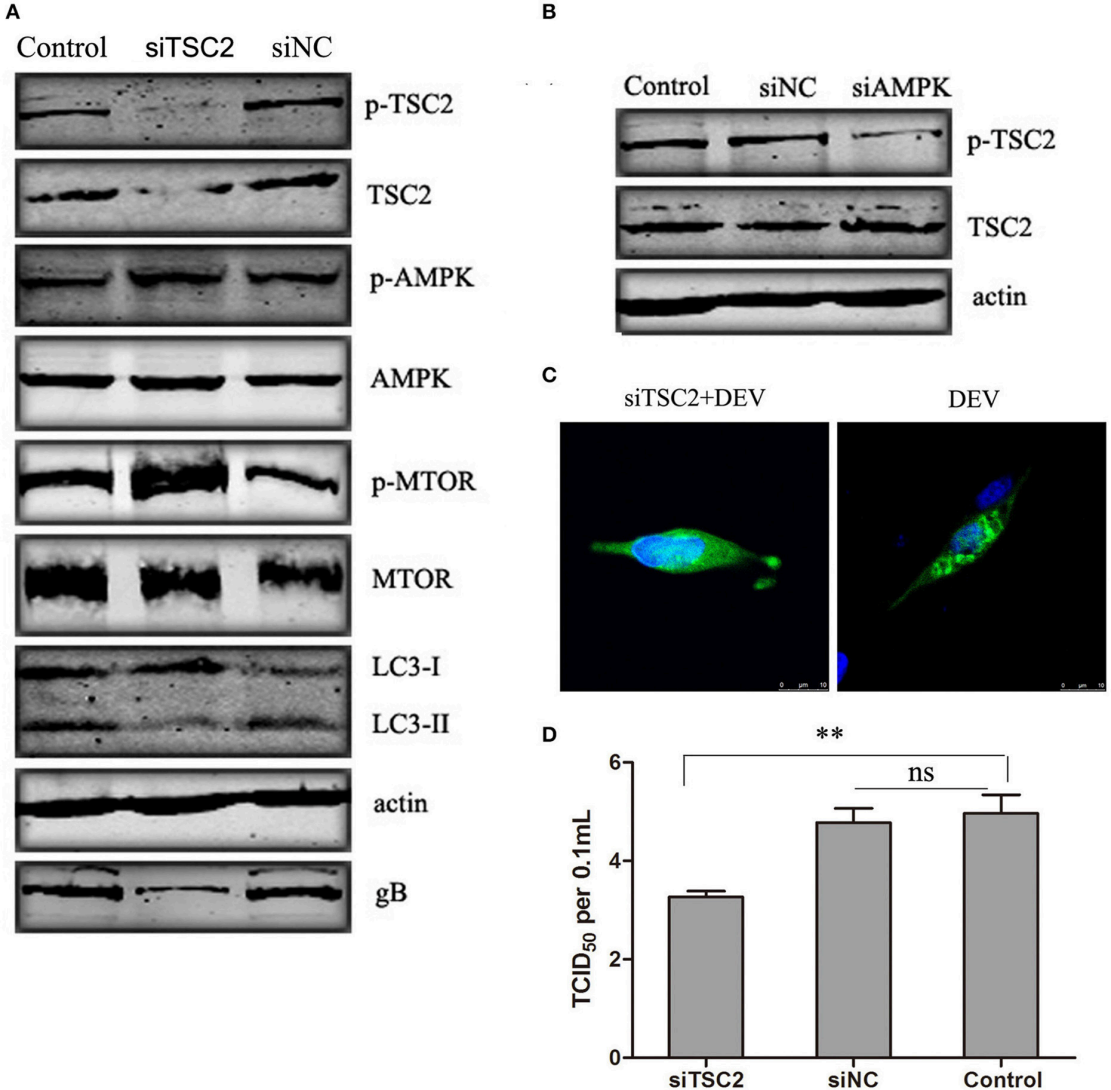
TSC2 is involved in AMPK–mTOR signaling pathway mediated DEV-induced autophagy. **(A)** Effects of TSC2 silencing on the signaling-pathway-related proteins in autophagy. DEF cells were transfected with TSC2 siRNAs or siNC for 24 h, then infected with DEV. At 48 hpi, expression of proteins was analyzed by western blotting. **(B)** Knockdown of AMPK affects the activity of TSC2. DEF cells were transfected with siAMPK or siNC for 24 h, and cells were then infected with DEV. At 48 hpi, TSC2 activity was analyzed by western blotting. **(C)** Representative confocal images of DEV with or without siTSC2 treatment for 48 h. DEF cells were transfected with GFP-LC3, along with siTSC2 or siNC for 24 h, then cells were treated with DEV for a further 48 h. GFP-LC3 puncta were analyzed. **(D)** The DEV yields produced by siNC- or siTSC2-transfected DEF cells were tested and shown as TCID_50_/ml at 48 hpi. The difference between two group means is presented as and ^**^P < 0.01.

TSC2 is an essential player in the coordination of cellular energy levels and cell growth or survival (Inoki et al., 2003). It has been shown that TSC2 is a coordinator of energy levels in cells, AMPK is a sensor of cellular energy and positively regulates TSC2, while MTOR is a downstream target of the TSC2-regulated cell response (Huang and Manning, 2009). To illustrate whether TSC2 is an essential component of AMPK–mTOR in DEV-infected cells, three pairs of siRNAs targeting TSC2 were designed, and the silencing effect was detected. The effective maximum of three siRNAs targeting TSC2 was TSC2-3# (Figure S5). The siRNA was transfected and proved to reverse the inhibition of MTOR and down-regulated LC3II expression, suggesting that TSC2 regulates autophagy via the MTOR pathway. Activation of AMPK had no obvious effect on cells transfected with TSC2-specific siRNA infected with DEV (Figure 5A). Similar results were obtained in cells treated with another two siRNAs (siTSC2-1#and siTSC2-2#) (Figure S10). We hypothesized that AMPK is an upstream regulator of TSC2 in DEV-mediated autophagy. To verify this speculation, we transfected cells with siRNA targeting AMPK, which inhibited activation of AMPK and TSC2 (Figure 5B). GFP-LC3 aggregation was markedly decreased in DEV-infected cells treated with TSC2-specific siRNA compared to cells treated with control siRNA (Figure 5C), which suggested that TSC2 was required for DEV-induced autophagy. Knockdown of TSC2 reduced replication of DEV through the disturbance of autophagy (Figure 5D). These results imply that TSC2 is a pivotal role of the AMPK–mTOR signaling pathway, which contributed to DEV-induced autophagy.

### Cell viability unaffected by pharmacological and siRNA treatment

In order to rule out case treatment with drugs or siRNAs could affect cell viability to inteference our results. The effects of the compounds used in this study on cell viability were detected using the WST-1 assay. The viability of the treated cells was almost equal to that of the untreated cells. So, we conclude that the drugs and siRNAs used in our study did not affect DEF viability.

## Discussion

Autophagy is a conservative biological process that involves lysosomal degradation of aging cell organelles, longevity proteins and exogenous pathogenic microorganisms, so as to achieve the recycling of energy. When cells are faced with a simultaneous exogenous stimulation, cells tend to activate the autophagy pathways. There is a lot of evidence indicate that many viruses induce autophagy, which plays an important role in their pathogenic mechanism and life cycle (Dreux and Chisari, 2010; Sir and Ou, 2010).

In our previous study, we found that DEV, a complex DNA virus, belonging to the family Herpesviridae can induce autophagy and facilitate its own replication (Yin et al., 2017). DEV activates autophagy; however, the mechanism for this is still not known. In this study, we found that impaired cellular energy metabolism contributed to DEV-induced autophagy via the AMPK–TSC2–MTOR signaling pathway.

Many virus infections that induce a cell response may create a hostile environment for the virus, which controls its replication and reduces the pathogenicity of the infection. Some viruses have developed strategies to reduce the cell response to ensure successful replication. In the mutual relationship between the host and viruses, changes in ATP production are typical (Lv et al., 2016). In our study, DEV infection decreased ATP production and caused mitochondrial ultrastructural alterations in DEF cells.

AMPK is a serine–threonine kinase that functions as a “fuel gauge” and maintains energy homeostasis during cellular stress (Qi and Young, 2015). AMPK is sensitive to the cytosolic AMP-to-ATP ratio, and metabolic stress activates autophagy through suppression of mTOR signaling (Shang and Wang, 2011; Tait and Green, 2012). In our study, the decrease in the ATP level was triggered by DEV infection, which participated in activation of AMPK and autophagy.

Many signal cascades are involved in the complex regulation of autophagy, in order to respond to different intracellular or extracellular stimuli. Similar to the system in yeast, the well-known serine/threonine kinase mTOR in mammalian cells, is a central regulator of autophagy and various other pathways. The data in this paper supports the understanding that mTOR is a regulator of autophagy in mammalian cells (Diaz-Troya et al., 2008; Jung et al., 2010; Li et al., 2015). mTOR activity is regulated by many upstream signals, such as PI3K/Akt, AMPK-related downstream regulated molecules and p53, which are involved in the initiation of autophagy. We have identified that AMPK may be involved in the mechanism of impaired cellular energy metabolism that contributes to DEV-induced autophagy. AMPK is a key regulator of cell metabolism and plays an important role in the process of autophagy. Phosphorylation of AMPK can negatively regulate the mTOR complex which can initiate autophagy (Shang and Wang, 2011). Similarly, we also showed that AMPK regulates DEV-induced autophagy through mTOR.

Phosphorylation activation of TSC1/2 activates autophagy by interfering with GTPase Rheb activity, which avoids mTOR activation by GTPase Rheb, thus inhibiting mTOR activity (Huang and Manning, 2009). We demonstrated that activation of AMPK by energy starvation results in direct phosphorylation of TSC2 in DEF cells infected with DEV, and inhibition of mTOR to initiate autophagy. Therefore, TSC2 is an important role of the AMPK–mTOR signaling pathway and is involved in the regulation of DEV-induced autophagy. This autophagy response is contributed to by the impaired cellular energy metabolism of DEV infected cells.

The two upstream signals of AMPK are cellular energy and Ca^2+^-mediated calcium/calmodulin-dependent protein kinase kinase (CaMKK)β . CAMKKβ and calcium are involved in AMPK activation in endothelial cells, T cells and hypothalamic neuron cells, indicating that calcium metabolism also plays a role in AMPK-MTOR-mediated autophagy regulation (Hoyer-Hansen et al., 2007). Recent research has shown that CaMKKβ is stimulated by an increase in the intracellular Ca^2+^ level and further activates AMPK; the latter potently induces autophagy in cells infected with rotavirus (Crawford et al., 2012). Thus, further research is needed to establish whether intracellular Ca^2+^ levels are triggered by DEV infection and participate in the activation of AMPK and induction of autophagy.

## Ethics statement

This study was carried out in accordance with the recommendations in the Guide for the Care and Use of Laboratory Animals of the National Institutes of Health, and approved Harbin Veterinary Research Institute. The animal Ethics Committee approval number is Heilongjiang-SYXK-2006-032.

## Author contributions

HC and LZ designed the experiments. HY and SL performed the experiments. HY and LZ wrote the paper. LX and YW analyzed the data and drew the graphs.

### Conflict of interest statement

The authors declare that the research was conducted in the absence of any commercial or financial relationships that could be construed as a potential conflict of interest.
