# Supplementary material for: Impaired Cellular Energy Metabolism Contributes to Duck-Enteritis-Virus-Induced Autophagy via the AMPK–TSC2–MTOR Signaling Pathway
Source: Front Cell Infect Microbiol. 2017 Sep 26;7:423. doi: 10.3389/fcimb.2017.00423 (PMC5622931; doi:10.3389/fcimb.2017.00423)
Supplement: Supplementary file 1 [file Image1.PDF]

Impair cellular energy metabolism contributes to duck-enteritis-virus-induced autophagy via the AMPK–TSC2–MTOR signalling pathway

*Haichang Yin#, Lili Zhao#, Siqi Li, Lijing Xu, Yiping Wang and Hongyan Chen**

Figure S1 Energy stress can contribute to DEV-induced autophagy in DEF cells
Figure S1 showed the raw uncropped images of figure2B

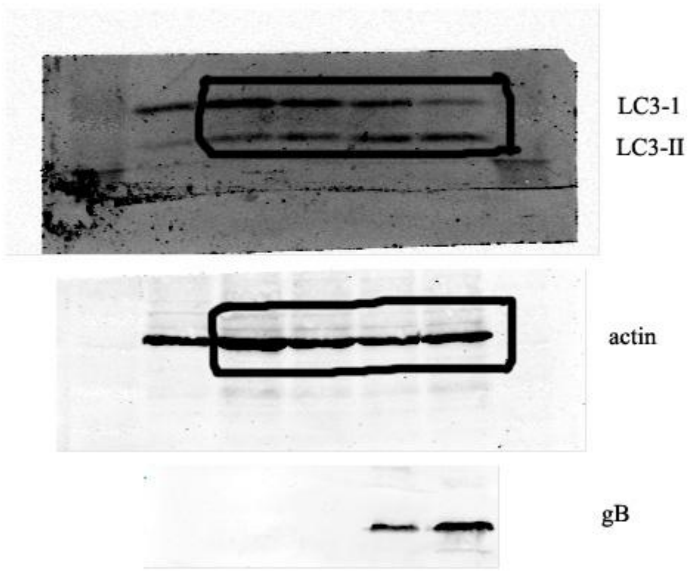

Figure S2 AMPK–mTOR may be involved in DEV-induced autophagy
Figure S2 showed the raw uncropped images of figure3A and3B
Figure3A

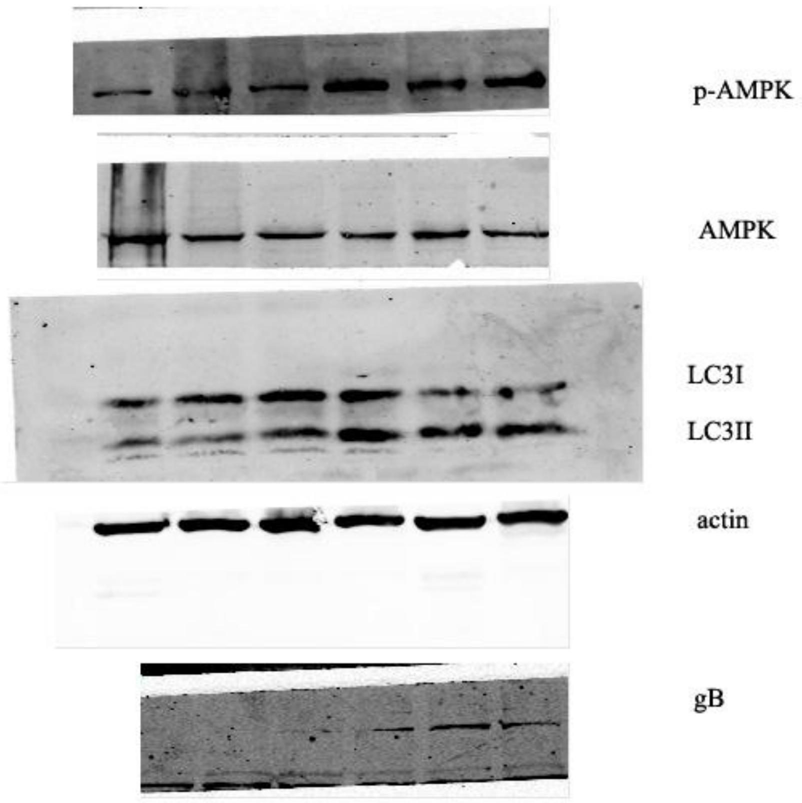

Figure3B

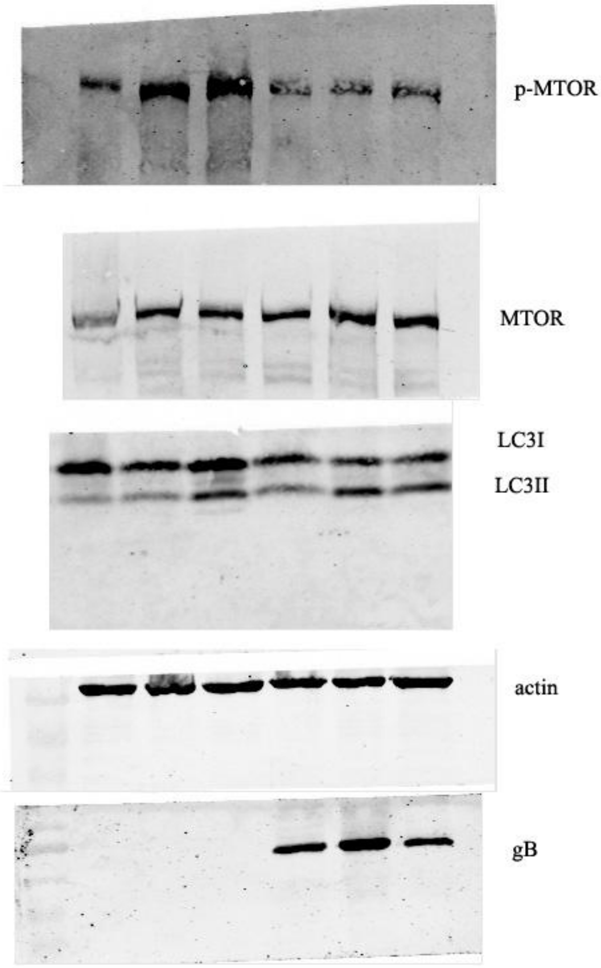

Figure S3 AMPK regulates DEV induced autophagy through MTOR
Figure S3 showed the raw uncropped images of figure4A and4B
Figure 4A

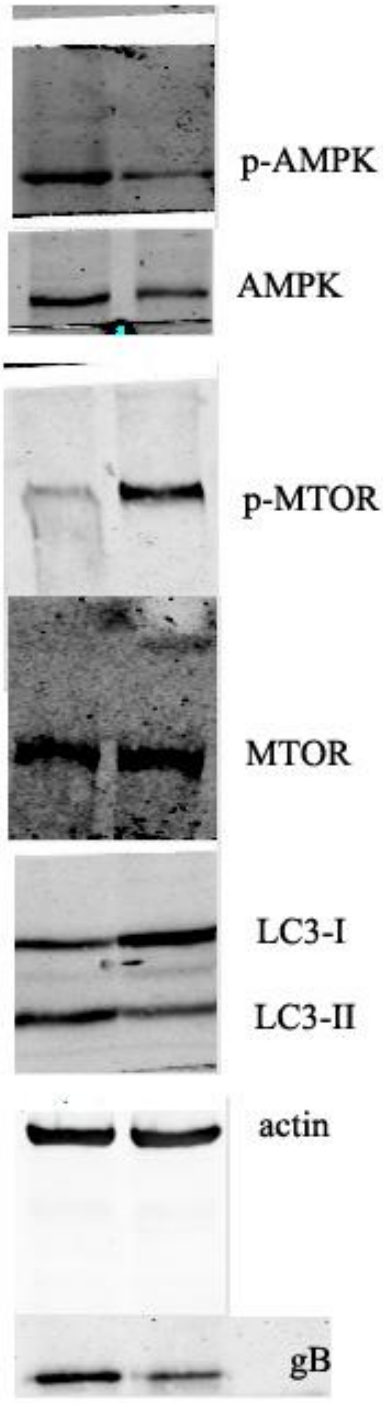

Figure4B

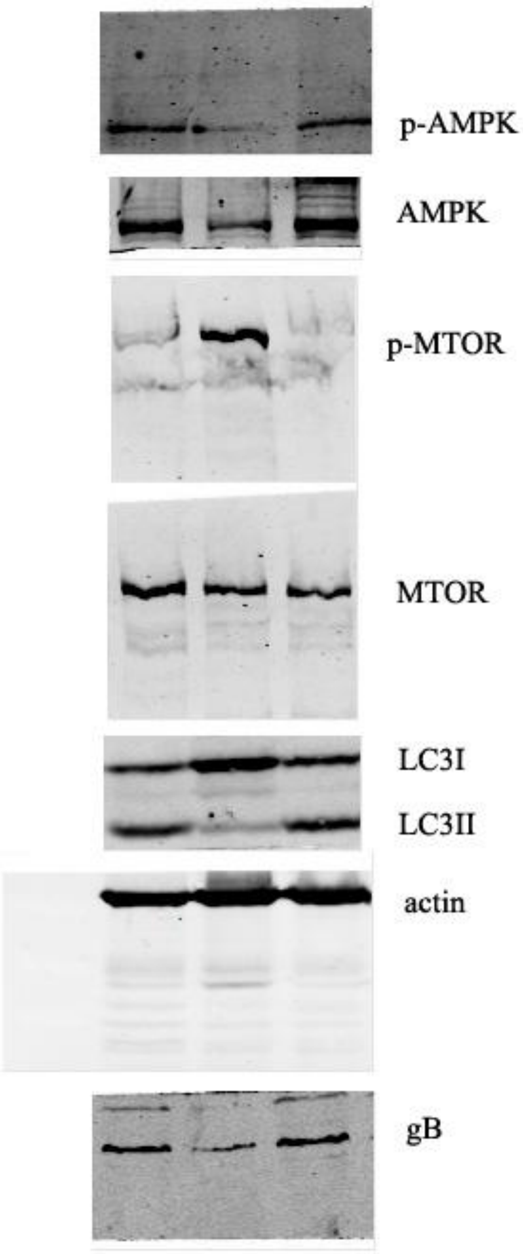
p-AMPK
AMPK
p-MTOR
MTOR
LC3I
LC3II
actin
gB

Figure S4 TSC2 is invovled in AMPK-MTOR signalling pathway mediated DEV induced autophagy

Figure S4 showed the raw uncropped images of figure 5A and 5B

Figure 5A

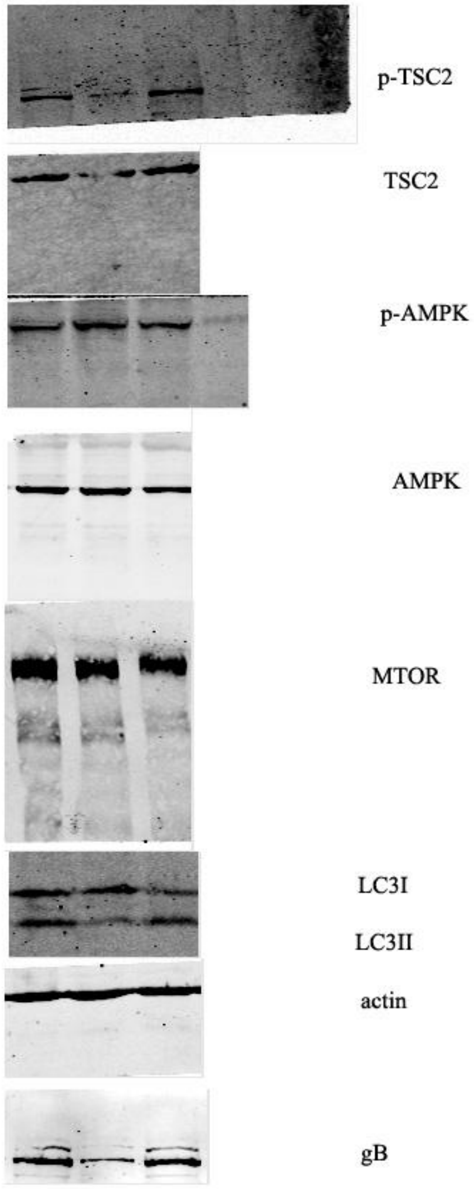

Figure5B

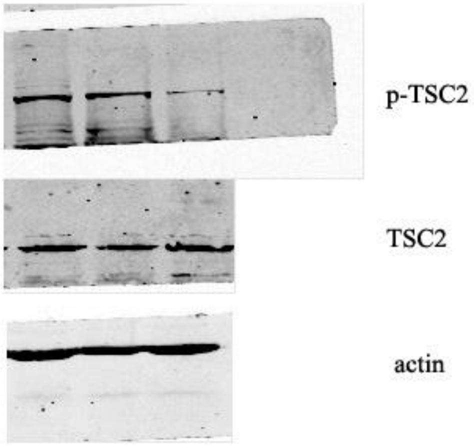

FigureS5 Western blotting analysis of the effectiveness of AMPK and TSC2 knockdown in DEF cells

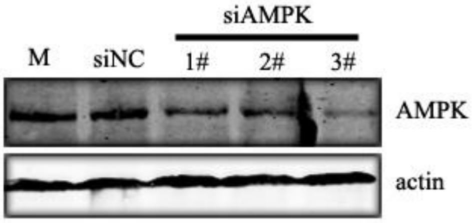

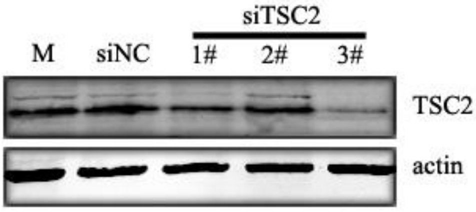

FigureS5 Western blotting analysis of the effectiveness of AMPK and TSC2 knockdown in DEF cells. DEF cells were transfected with control siRNA (siNC) or AMPK-specific siRNAs and TSC2-specific siRNAs for 24 h and then analyzed by Western blotting

FigureS6 ATP production is inhibited by chemicals oligomycin(5μM), indeed enhance autophagy and DEV replication.

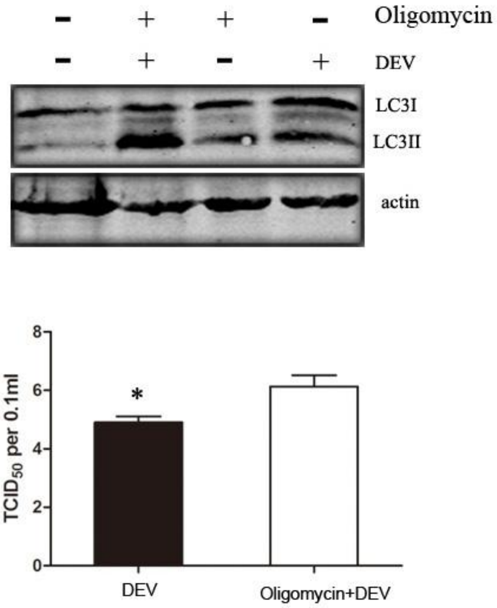

FigureS6 ATP production inhibitor could enhance DEV replication and autophagy. Mock- or DEV-infected DEF cells were treated with 5μM oligomycin for 36 h or untreated, and the ATP levels were measured. Cells were infected and treated, and at 48 hpi, cell samples were analysed by immunoblotting with antibodies against LC3B and β-actin. Increase of DEV replication by oligomycin treatment. Cells were pretreated and infected. At 48 hpi, virus titres were measured using the $TCID_{50}$ assay.

FigureS7 DEV infeciton can not effect cell viability

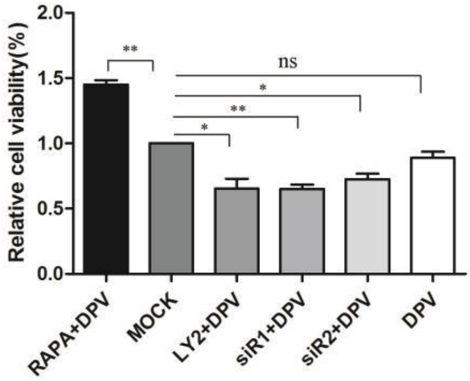

FigureS7 DEV infeciton had no effect on cell viability. After cells were treated with DEV for 48 hours, cell viability was tested by WST kits as absorbent density at 450 nm expressed as relative cell viability (ratio of treated to blank cells). Bar represents ±SD; ns indicates no significant difference, $p>0.05$.

FigureS8 Band intensity of p-AMPK and AMPK to confirm the compound C induced p-AMPK reduction is not a result of reduction of total AMPK level

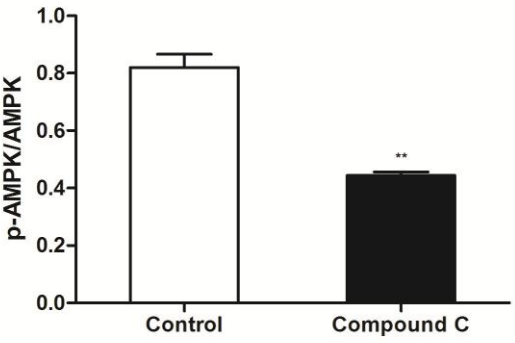

FigureS8 Intensity band ratio of p-AMPK to AMPK. Effects of Compound C treatment on LC3II, phosphorylation and total levels of AMPK. Cells were pretreated with Compound C (5 μM) or DMSO (control) for 1 h, followed by DEV adsorption for 2 h. At 48 hpi, the protein levels were measured by western blotting. Intensity band ratio of p-AMPK to AMPK.

FigureS9 The effects of another two siRNAs AMPK respectively on mTOR,autophagy and viral yield.

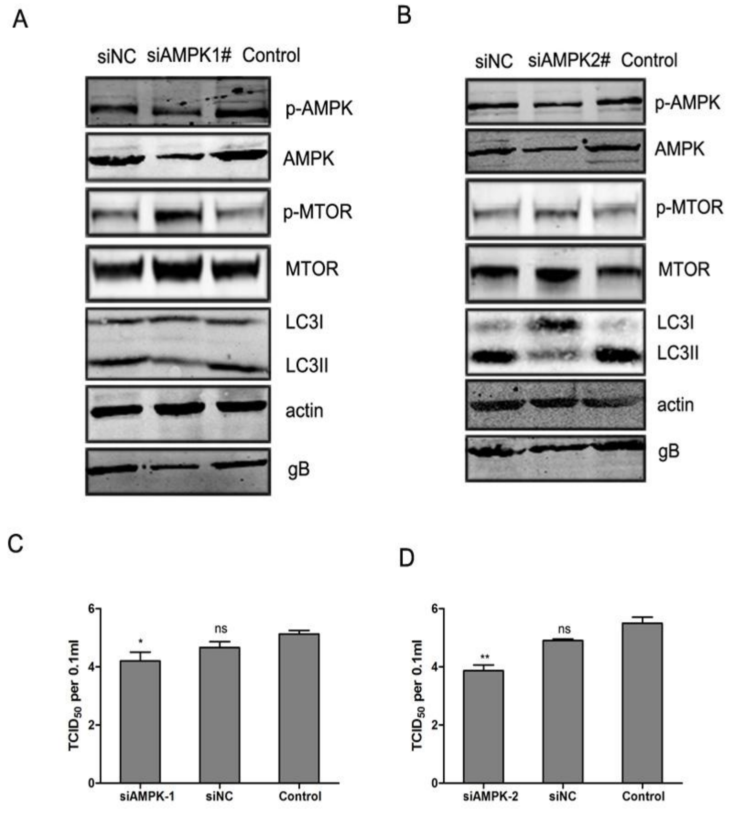

FigureS9 The effects of another two siRNAs AMPK respectively on mTOR,autophagy and viral yield. (A,B) DEF cells were transfected with AMPK-specific siAMPK-1# and siAMPK-2#, then infected with DEV. At 48 hpi, cells were harvested and western blotting was performed. **(C,D)** Virus yields in DEF cells transfected with siAMPK-1# and siAMPK-2# . Virus titres were measured using the $TCID_{50}$ assay.

FigureS10 The effects of another two siRNAs TSC2,respectively on mTOR,autophagy and viral yield.

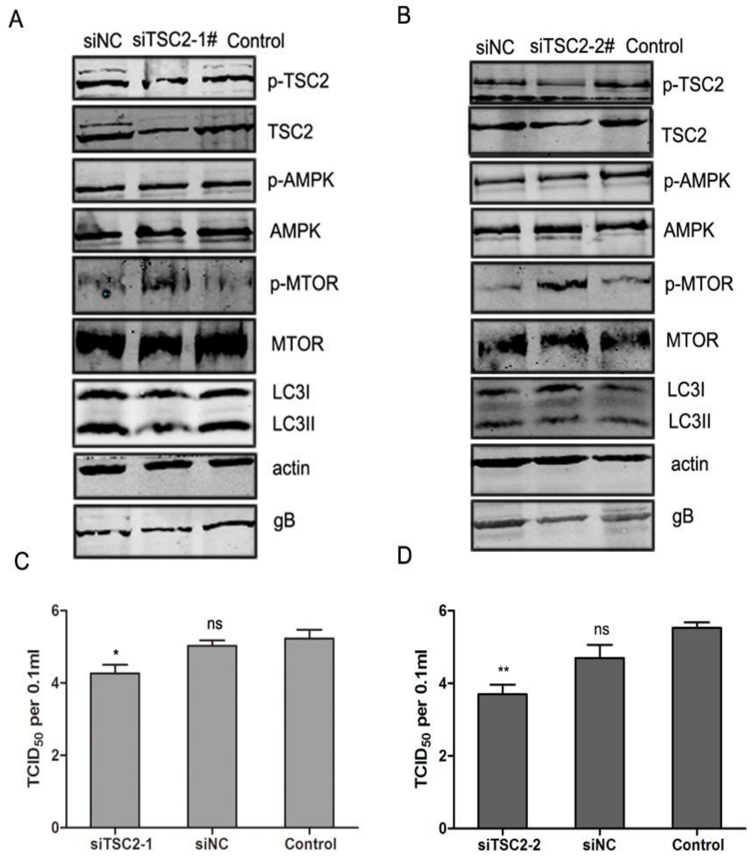

FigureS10 The effects of another two siRNAs TSC2 respectively on mTOR,autophagy and viral yield. (A,B) DEF cells were transfected with TSC2-specific siRNA-1# and siRNA-2#, then infected with DEV. At 48 hpi, cells were harvested and western blotting was performed. **(C,D)** Virus yields in DEF cells transfected with siTSC2-1# and siTSC2-2# . Virus titres were measured using the $TCID_{50}$ assay.
